# Audience-Customized Translation of Rule-Based Evidence with Large Language Models Across Multiplexer Benchmarks

**DOI:** 10.1145/3795101.3814722

**Published:** 2026-08-13

**Authors:** Harsh Bandhey, Gabriel Lipschutz-Villa, Khoi Dinh, Malek Kamoun, Ryan J. Urbanowicz

**Affiliations:** Cedars Sinai Health Sciences University, Los Angeles, CA, USA; University of Pennsylvania, Philadelphia, PA, USA; University of Pennsylvania, Philadelphia, PA, USA; University of Pennsylvania, Philadelphia, PA, USA; Cedars Sinai Health Sciences University, Los Angeles, CA, USA

**Keywords:** interpretable machine learning, rule-based learning, natural language explanations, large language models, explainable AI, evaluation

## Abstract

Evolutionary rule-based machine learning (ERBML) algorithms can capture complex relationships while still yielding highly interpretable models comprised of IF:THEN rules. During prediction, ‘matching’ rules contribute to, and form the explanation for, the model’s prediction. However, rules and their associated parameters (in their raw form) are likely too technical for their intended users. This study examines the feasibility of using a large language model (LLM) to translate the prediction evidence from matching rules into natural language text for different audiences, e.g. layman, clinician, expert. Using models trained by the ‘HEROS’ ERBML on MUX benchmarks, we evaluate LLM text quality metrics under different scenarios (i.e. 1,800 prediction explanations). We observe that (1), intuitively, LLM quality performance improves on HEROS models that have been more ideally trained, (2) making a glossary available to the LLM to define feature names generally raises explanation *audience-fit* scores and sometimes lowers *overstatement* (beyond rule-evidence), but it also lengthens explanations and often increases *hallucination rate*, and (3) audience customization creates some LLM performance trade-offs. These results suggest that constrained LLM translation of rules for natural language prediction explanations is feasible, while highlighting the importance of carefully designing the LLM prompts and evidence input from the ERBML.

## 1 Introduction

Interpretable machine learning (ML) is especially valuable in settings where model behavior must be inspected, challenged, or communicated across user groups with different technical backgrounds. Evolutionary rule-based machine learning (ERBML) is attractive in this context because it can represent predictions through explicit IF–THEN rules rather than through opaque latent parameters. During prediction, the subset of rules in the selected model whose conditions are satisfied by the current instance form the *matching rules*. Throughout the paper, *prediction-time* evidence denotes the post-training, per-instance information produced when a selected rule model is applied to a specific instance: the matching rules, class prediction, class probabilities, and associated metadata. Those matching rules provide the prediction-time evidence that the present study seeks to verbalize. HEROS (Heuristic Evolutionary Rule Optimization System) is one such ERBML system. It separates optimization of individual rules from optimization of rule sets (as models), yielding a compact predictive rule-based model with prediction-time evidence from matching rules [15]. More broadly, HEROS belongs to a learning-classifier-system and rule-based ML lineage that emphasizes explicit rule structure and model inspectability [23-25]. Subjectively, raw rules and their prediction-time metadata are not necessarily ‘interpretable’ for clinicians, patients, or other non-expert readers that may lack the ML or statistical background needed to put raw rule explanations into the proper context for understanding. A natural language response that provides necessary context would likely be preferable.

Large language models (LLMs) offer a natural mechanism for improving the presentation of such evidence, and related work has explored natural-language explanations of model predictions through model-agnostic and pipeline-based approaches [18, 28]; however, recent work on natural-language explanations has shown that fluent text can be only weakly coupled to the reasoning process that produced a prediction [1, 17, 21]. In high-stakes settings, this gap is particularly problematic because readers may mistake an explanation of model behavior for a causal explanation of the world itself [5]. These concerns motivate a narrower objective than generic explanation generation. Rather than asking an LLM to infer reasons from raw data or to rationalize a black-box predictor post hoc, we ask it to translate evidence that has already been produced by an interpretable model. This framing is related to hybrid work that combines LLMs with symbolic or rule-based structure rather than leaving reasoning fully unconstrained [22, 26, 30]. The present setting differs in one important respect: the rule-based classifier produces the explanation evidence first, and the LLM is used only to restate that evidence for a specified audience.

This preliminary study examines three related questions. First, how strongly does downstream explanation quality depend on the quality of the learned HEROS rule model that supplies the prediction-time evidence? Second, when the prediction-time evidence is fixed, what changes when the prompt adds a glossary of feature names? Third, when the same prediction-time evidence is restated for layman, clinician, and expert readers, what are the performance trade-offs? These questions are examined by varying the HEROS training profile, prompts, and specified target audience.

We conduct an initial six-run study using multiplexer benchmark datasets (i.e. MUX6, MUX11, and MUX20). The central premise is that explanation quality is jointly shaped by three comparison levels: upstream model quality (minimal versus extensive HEROS training), prompt design (*evidence-only* versus *evidence+glossary*), and audience target (*layman, clinician*, and *expert*). We seek to evaluate resulting LLM model explanations using a broad selection of previously defined and proposed evaluation metrics (defined below) including hallucination rate, causal overclaim rate, evidence precision, evidence recall, etc. The primary contribution of this work is to establish the feasibility of an audience-conditioned natural-language translation layer for ERBML prediction-time evidence. This contribution reasonably assumes that natural language explanations are preferable over raw rule representations for most target audiences in real-world applications. While the HEROS algorithm is used in this study, our proposed LLM explanation approach could potentially be utilized in combination with other rule-based ML algorithms.

## 2 Methods

The experimental design corresponds directly to the three study questions. HEROS supplies the evidential content so that the LLM is never required to infer explanations from raw inputs alone. The first comparison level varies HEROS training budget and therefore the quality of the learned rule model that supplies the prediction-time evidence packet. The second varies prompt design through the presence or absence of a minimal glossary. The third varies the intended audience and therefore the level and style of explanation requested from the LLM. Evaluation then separates evidence preservation, unsupported addition, and audience fit.

### 2.1 HEROS and Rule-based Machine Learning

Interpretable rule-based ML spans several related traditions. Classical rule-induction systems such as RIPPER learn compact ordered IF–THEN rules directly from labeled data [6], whereas evolutionary rule learners such as BioHEL search for readable rule sets through genetic optimization [2]. Within learning classifier systems, XCS [27] and UCS [4] established an accuracy-based formulation in which predictions are made from matched rules and fitness favors accurate, general classifiers rather than only raw reward or coverage. More recent supervised Michigan-style systems such as ExSTraCS [25] adapted this lineage to noisy, heterogeneous biomedical settings while preserving explicit rule representations [23, 25]. This study utilized HEROS [15], a ERBML algorithm inspired by ExSTraCS, but which sought to discover maximally compact rule-sets without relying on a default rule (like RIPPER and BioHEL). HEROS was chosen specifically because we expect an LLM explanation system to be more effective when combined with algorithms (1) yielding maximally compact rule sets (since fewer rules are expected to match a given instance to be explained) and (2) that do not rely on a default rule (which inherently lack direct explanations for instances predicted by the default rule). Like XCS, UCS, and ExSTraCS, HEROS remains a Michigan-style evolutionary rule learner: rules are maintained in a population, updated through crossover and mutation, and evaluated by accuracy- and coverage-oriented signals rather than raw frequency alone.

HEROS is distinguished by its two-phase training design [15]. In Phase 1, HEROS evolves an interpretable rule population and evaluates individual rules with a Pareto-inspired objective that favors accurate rules covering informative subsets of the training data. In Phase 2, HEROS treats candidate predictive models as subsets of those learned rules and evolves rule sets under a separate multi-objective search that prioritizes predictive performance while penalizing unnecessarily large models. The result is a population of candidate rule sets together with the rules from which they are assembled. At prediction time, one selected rule set is applied to a new instance; the rules in that model whose conditions match the instance form the match set, and those matching rules generate the prediction array used for class prediction. This separation between rule learning and rule-set learning is central to the present study because it exposes both a compact predictive model and prediction-time evidence from matching rules. We evaluate two HEROS training profiles. The *minimal* profile uses 10,000 rule-learning iterations, a rule population of 500, and 40 model-level iterations. The *extensive* profile uses 100,000 rule-learning iterations, a rule population of 1,000, and 100 model-level iterations. These profiles should be interpreted as lower- and higher-budget HEROS training conditions rather than as a guarantee that the higher-budget condition recovers an ideal model on every benchmark. In all runs, Group and InstanceID are excluded from model training and retained only for split management and logging.

### 2.2 From a HEROS Model to a Prediction

The explanation pipeline begins only after HEROS has been trained. For each benchmark, HEROS is fit on the training split, producing a learned rule population and a learned population of candidate rule sets. The explanation system then selects one trained rule set to serve as the predictive model and uses only that model when explaining held-out instances. The LLM therefore translates the prediction-time evidence produced by one selected learned HEROS model rather than an idealized multiplexer program or the full evolved rule population.

In HEROS, a predictive model is itself a rule set rather than a single parametric object. Phase 1 evolves individual rules, and Phase 2 evolves candidate models by selecting subsets of those rules and evaluating them as complete rule sets [15]. At prediction time, HEROS applies the best discovered model (i.e. rule-set) to make predictions. Prediction begins by scanning that rule set to construct the match set *M*, defined as the subset of rules whose IF conditions match the current instance. Throughout the paper, these are the *matching rules*. HEROS then aggregates those matching rules into a class-wise prediction array.

For classification, each matching rule *r* ∈ *M* contributes its empirical class distribution *p_r_*(*c*) together with its numerosity *n_r_*. HEROS computes the unnormalized class vote

$$V(c)=\sum_{r\in M}n_{r}p_{r}(c),$$

then normalizes by the total numerosity in the match set,

$$P(c)=\frac{V(c)}{\sum_{r\in M}n_{r}}.$$

The predicted class is the class with the largest *P*(*c*). If there is a tie, HEROS breaks it using the training majority among the tied classes, and if that is also tied it falls back to random selection. If no rules match, the instance is uncovered and HEROS applies its default uncovered-case behavior. In ‘muliplexer’ benchmark problems, the address bits *A* point to one of the register bits *R_k_*, and the class equals the value of that selected register bit. In the ideal MUX6 rule set, an instance with *A*_0_ = 1, *A*_1_ = 0, and *R*_2_ = 1 is covered by a single matching rule, yielding *P*(0) = 0.0 and *P*(1) = 1.0. Supplementary Table S4 gives the full ideal MUX6 rule set together with a worked match-set and probability calculation. Learned HEROS models are typically less minimal, which is why the downstream translation problem becomes harder as match sets become denser.

### 2.3 LLM Model Selection

All reported generations and judge scores were produced through the OpenAI API with gpt-4.1-mini. This model was selected because the task is structured, bounded, and repeated at scale across datasets, training profiles, prompt experiments, audiences, and judge passes. Under this workload, a lower-latency instruction-following model is a practical fixed choice for this study rather than introducing further experimental variables [19, 20]. Generations used temperature 0.0 and a 400-token limit. Judge scoring used the same model family at temperature 0.0 with a 300-token limit.

### 2.4 Pipeline Design

The pipeline is defined by a strict division of labor between HEROS and the language model. HEROS performs prediction and identifies the matching rules for a given instance. The LLM receives only a structured prediction-time evidence packet derived from those outputs. It is not asked to summarize the full rule population, assign new semantics to feature names, or add reasons beyond the packet supplied by HEROS.

For each held-out instance, the system assembles a prediction-time evidence packet containing the predicted class, class probabilities, model coverage status, matching rules partitioned into supporting and contradictory rules, relevant instance feature values, and a short model-context summary. When available, the packet also includes rule metadata such as numerosity, accuracy, fitness, vote contribution, and training-support counts. Internal feature indices are converted to feature names before prompt construction. Each matching rule is then rendered as readable IF–THEN text so that the generator receives a textual but still structured representation of model evidence.

Prompt construction follows the same constrained-translation philosophy. We compare two experimental prompt designs. An *evidence-only* prompt provides prediction output, matching rules, and relevant instance values. An *evidence+glossary* prompt adds concise one-sentence glossary entries for features appearing in matching rules. These glossary entries were intentionally minimal and feature-oriented rather than mechanism-oriented, because a more detailed glossary could leak the true multiplexer mechanism into the prompt. The audience setting determines how much technical detail is exposed. Layman explanations use plain language with qualitative confidence wording only. Clinician explanations use concise feature–value reasoning with moderate technical detail and rounded support information. Expert explanations include exact probability language, rule identifiers, and metadata when available. Across all settings, the prompt instructs the LLM to discuss model behavior only, acknowledge conflicting rules when present, and avoid causal or factual claims that extend beyond the provided evidence.

Supplementary Tables S1 and S2 summarize the shared system prompt, audience-specific add-ons, prompt-condition differences, and judge rubric used in the final study. Runnable implementations are in evaluation/experiments/heros_llm/, including prompt_templates.py, prompt_builder.py, and judge.py.

### 2.5 Benchmarks and Experimental Design

We evaluate on the multiplexer benchmarks distributed with the HEROS repository wherein a target number of MUX instances were generated. All MUX datasets involve binary feature values and outcomes. The MUX6 dataset included 500 total instances split into 450 training and 50 held-out test cases. Of note, for the MUX6 problem, only 2^6^ = 64 possible binary input patterns are possible, but 500 total are generated (with repeated instances) to sample from for training/testing split purposes. The MUX11 dataset contained 5,000 total instances split into 4,500 training and 500 held-out test cases. The MUX20 dataset contained 10,000 total instances split into 9,000 training and 1,000 held-out test cases. HEROS is trained on the standard training split for each benchmark. For MUX6, LLM explanation evaluations used the full 50-instance held-out test fold. For MUX11 and MUX20, this evaluation used stratified 50-instance subsets drawn from the held-out test folds in order to keep the LLM evaluation budget fixed across datasets while preserving variation in predicted class and rule-matching profile.

Both HEROS training profiles are run on each benchmark. This design yields 50 evaluated instances per dataset, two training profiles, two experimental prompt designs, and three audience targets, for a total of 1,800 explanations. The minimal and extensive profiles should therefore be read as lower- and higher-budget HEROS training conditions within the present study, not as a guarantee that the extensive profile reaches an ideal solution on every benchmark. Supplementary Tables S3-S6 document the benchmark dataset counts, the exact HEROS and run-parameter settings used to produce these runs, and an illustrative example of the ideal-MUX6 solution. The LLM is evaluated against the prediction-time evidence packet produced by the learned HEROS model rather than against the ideal benchmark logic, because the downstream task is to restate what the trained model actually used for that prediction. The goal is to evaluate model-faithfulness (i.e. what the trained model specified) rather than a ground-truth causal-explanation (e.g. what a domain expert would regard as the true explanation of the outcome). Deterministic settings were used throughout to reduce sampling variance and to support fully logged API-based experiments.

### 2.6 Evaluation Metrics

Table 1 summarizes and defines all metrics utilized in this paper. The evaluation framework uses two complementary layers of metrics, a faithfulness-and-safety layer and an audience-fit layer. The faithfulness-and-safety metrics test whether the explanation preserves prediction-time evidence and avoids unsupported additions. Audience-fit metrics test whether that same evidence is understandable and technically appropriate for the intended audience. Higher *evidence recall* and *rule coverage* indicate better evidence preservation, whereas higher *hallucination rate* and *causal overclaim rate* indicate less controlled verbalization. In this study, *hallucination rate* refers to unsupported additions in LLM explanations, whereas causal overclaim rate refers to wording that overstates model reasoning as causal or factual truth. Higher *audience understandability* and *audience technical fit* indicate better audience fit, but these remain proxy signals rather than participant-based outcomes. *Flesch reading ease, Flesch–Kincaid grade level*, and *word count* are treated as descriptive surface-form measures rather than direct judge-based audience-fit metrics.

The audience-fit layer is necessary because faithfulness-and-safety metrics alone cannot determine whether an explanation is understandable or pitched at the appropriate technical level for a given audience. The *audience understandability* and *audience technical fit* metrics in the audience-fit layer use an LLM to evaluate LLM-generated text, which is a methodological limitation, particularly because the generator and judge come from the same model family [12, 16]. These should thus be interpreted cautiously rather than as human-grounded evidence in the sense of Doshi-Velez et al. [8] and are only treated as scalable proxies for human participant-based evaluation.

## 3 Results

The results are organized around the three comparison levels introduced earlier. HEROS training profile, prompt design, and audience target. We begin with dataset-level training-profile effects, then illustrate those contrasts in a single MUX11 case, and finally aggregate the prompt and audience comparisons across datasets.

### 3.1 Effects of HEROS Training Profile

Table 2 isolates the training-profile comparison, Table 3 gives a qualitative case, and Tables 4 and 5 aggregate prompt and audience effects. Across all six runs, *evidence precision* and *prediction–explanation agreement* remained perfect and are omitted from the tables. Lower mean matching-rule count implies a smaller evidence packet and therefore a lighter compression burden for the LLM. Averaged across datasets, the *extensive* profile raises mean HEROS test accuracy from 80.4% to 87.3%, increases the ideal-rule fraction from 39.6% to 62.5%, and lowers mean matching rules from 6.07 to 5.37, but Table 2 shows that this aggregate pattern masks strong dataset-specific differences.

The three comparison levels play different roles in the downstream explanation task. HEROS training profile changes the quality and density of the evidence packet, and lower mean matching-rule count matters because it implies a smaller packet and therefore a lower compression burden for the LLM. Prompt experiment changes how much the LLM elaborates on that packet, so longer outputs together with lower readability and changing audience-fit scores indicate a shift in verbal detail, whereas *hallucination rate* and *causal overclaim* rate indicate whether that elaboration remains controlled. Audience target changes which failure mode becomes most visible: expert outputs preserve the most evidence, layman outputs are the safest under the hallucination and overclaim checks, and clinician outputs remain the most causally overstated.

MUX6 provides the simplest scenario. Both HEROS profiles recover an ideal 8-rule model, achieve HEROS test accuracy 1.000, and average exactly one matching rule per held-out instance. Under these conditions, downstream explanation coverage is perfect in both runs: *evidence recall* and *rule coverage* remain 1.000. The remaining differences are modest wording effects rather than evidence differences. The *minimal* profile yields a slightly lower *hallucination rate*, 16.0% versus 18.3%, and slightly higher audience scores, but the two MUX6 runs are substantively similar because the learned rule model is already ideal. MUX6 therefore serves as the control case for the remainder of the study: once the packet is minimal, the residual variation primarily reflects language-level choices. Consistent with this interpretation, *Causal Overclaim Rate* remains 0.0 in both MUX6 runs.

MUX11 is the clearest observed positive case for stronger HEROS training. Moving from the *minimal* profile to the *extensive* profile raises HEROS test accuracy from 81.2% to 98.8%, reduces the selected top model from 56 rules to 24 rules, increases the ideal-rule fraction from 18.8% to 87.5%, and lowers the mean number of matching rules from 4.86 to 2.24. These upstream changes coincide with downstream gains: *evidence recall rises* from 94.4% to 99.4%, *rule coverage* reaches 100.0%, *causal overclaim rate* falls from 17.0% to 9.0%, *audience understandability* and *audience technical fit* both improve slightly, and outputs become markedly shorter, from 126.11 words to 102.73. The main countervailing result is that *hallucination rate* rises from 5.7% to 18.3%. Even with that increase, MUX11 remains the clearest observed positive case in this study because training quality, evidence preservation, overclaim control, and output compactness move in the same overall direction.

MUX20 provides a useful boundary case for interpreting the training-profile comparison. The higher-budget HEROS profile raises HEROS test accuracy only slightly, from 60.1% to 63.2%, still recovers no ideal rules, and does not simplify the packet. Mean matching rules increase slightly from 12.34 to 12.88, *evidence recall* falls from 73.7% to 68.6%, and *rule coverage* falls from 94.3% to 93.0%. At the same time, *hallucination rate* drops from 12.0% to 8.7% and the audience-oriented scores improve slightly, but *causal overclaim rate* rises from 32.0% to 34.0%. In this benchmark, the metric directions no longer align: lower hallucination does not coincide with higher recall or lower overclaim. This pattern is also consistent with denser packets arising from overlapping, overgeneral, or overspecific rules, although the present study does not isolate the separate effects of specified-variable count, rule overlap, and rule-set size. Under the budgets tested here, the higher-budget HEROS profile helps MUX20 only modestly at the classifier level and does not translate into better evidence preservation or safer wording overall. MUX20 therefore serves as a useful boundary case in the present study: additional upstream search budget can improve classifier accuracy without yet making the explanation packet easier to translate. Taken together, the six-run comparison suggests that stronger HEROS training improves downstream explanation quality only when it also produces a cleaner and more ideal rule model.

### 3.2 Illustrative MUX11 Case

After the dataset-level comparison, Table 3 illustrates the three study axes within a single held-out case. We fix the prompt to *evidence+glossary* and use InstanceID 287 from MUX11 because the same held-out instance yields different HEROS packets under the two training profiles, all three audience versions remain readable, and the minimal-profile block exhibits a clear audience-separated hallucination pattern. Under the *minimal* profile, the expert output is flagged by the hallucination detector, whereas the layman and clinician outputs are not. This flag should be interpreted as a heuristic wording issue rather than as evidence that the model invented a new rule or feature.

The qualitative case is useful because it makes the packet-level and audience-level interactions visible in a way that aggregate tables cannot. Read vertically, the table shows how the same packet is restated differently for layman, clinician, and expert readers, trading brevity against detail and safety against completeness. Read horizontally, it shows how the learned HEROS packet itself changes the downstream task: the minimal packet is unanimous but weaker and denser, whereas the extensive packet is shorter, more structured, and explicitly conflict-bearing. The example is therefore not intended as a representative average case, but as a controlled illustration of how upstream model quality and downstream audience conditioning interact within the same held-out instance.

### 3.3 Cross-Dataset Prompt-Experiment Comparison

Table 4 addresses the prompt-experiment comparison by aggregating across datasets within each HEROS profile. *Evidence+glossary* prompt design behaves as a trade-off rather than a uniform improvement. Under the *minimal* profile, it raises *evidence recall* from 88.6% to 90.2% and *rule coverage* from 97.7% to 98.4%, but it also raises *hallucination rate* from 10.2% to 12.2% and lengthens the outputs from 116.28 to 121.47 words. Under the *extensive* profile, *evidence recall* changes little, from 89.2% to 89.4%, and *causal overclaim rate* falls from 15.8% to 12.9%, but *Hallucination Rate* rises from 14.2% to 16.0% and output length rises from 109.10 to 113.67 words. These shifts suggest that the glossary primarily encourages more explicit restatement of packet content rather than improving evidence selection itself. Overall, *evidence+glossary* prompt design promotes fuller but less controlled verbalization of packet evidence.

The readability metrics sharpen that interpretation rather than replacing it. Under both HEROS profiles, *evidence+glossary* prompt design slightly lowers *Flesch Reading Ease*, raises *Flesch–Kincaid Grade Level*, and lengthens the outputs, indicating that the added glossary information mainly encourages more elaborated text. The modest audience-fit gains therefore appear to come from fuller verbalization rather than from cleaner evidence selection. This is why the prompt comparison should be read as a presentation tradeoff: the glossary can make explanations feel more complete and sometimes more appropriate, but it also creates more opportunities for unsupported elaboration.

### 3.4 Cross-Dataset Audience Comparison

Table 5 then addresses the audience comparison by aggregating across datasets within each HEROS training profile. Across both profiles, *layman* prompting produces the lowest *hallucination rate*, the lowest *causal overclaim rate*, and the shortest outputs, but also the lowest *evidence recall*. Under the *minimal* profile, for example, layman outputs average 82.97 words with 2.7% hallucination and 4.3% *Causal Overclaim Rate*, but only 86.6% *Evidence Recall. Clinician* prompting lies between those extremes on recall and audience fit, but its *causal overclaim rate* is the worst of the three audiences, reaching 32.3% under the *minimal* profile. *Expert* prompting yields the highest *evidence recall* and the strongest audience-fit scores, but it also has by far the highest *hallucination rate*; under the *minimal* profile, expert outputs reach 93.0% *evidence recall*, 28.0% *hallucination rate*, and 152.16 words. This matters because explanation presentation is not neutral: the safest explanations and the most complete explanations are not the same object. This mirrors the dataset-level result: no single metric fully captures the trade-off.

Comparing training profiles within each audience shows that stronger HEROS training does not remove this trade-off. Under the *extensive* profile, layman outputs remain the safest, clinician outputs remain the most prone to overstatement, and expert outputs remain the most evidence-complete. Stronger training shortens the clinician and expert outputs and slightly lowers *causal overclaim rate* in those two settings, but it does not eliminate the expert setting’s tendency toward unsupported elaboration.

The readability metrics again function mainly as surface descriptors. Clinician outputs are the hardest by *Flesch reading ease* and *Flesch–Kincaid grade level*, whereas expert outputs score as easier by those formulas despite being more technical, confirming that readability formulas alone do not validate audience fit. This is one reason the paper keeps both metric layers: faithfulness-and-safety metrics track what evidence survives translation, whereas audience-fit metrics estimate whether the same content is pitched appropriately for the requested reader.

## 4 Discussion

The six-run comparison suggests that the learned rule model is part of the explanation system itself: the prediction-time evidence packet supplied by HEROS determines what the LLM can preserve, omit, or elaborate upon. The most stable result across all runs is that *Evidence Precision* and *Prediction–Explanation Agreement* remain perfect. The dominant risk is therefore not arbitrary invention of feature evidence, but variation in how completely the packet is preserved and how cautiously it is verbalized. The contribution should accordingly be read as a proof-of-concept translation layer for ERBML prediction-time evidence rather than as a new ERBML learner or a demonstration that an LLM is uniquely necessary for explanation presentation. Direct displays of the raw rule packet and deterministic rule-to-text verbalizers remain natural non-LLM baselines, but they are outside the scope of the present paper.

Upstream model quality receives a conditional answer. MUX6 is the control case, MUX11 is the clearest positive case, and MUX20 is the boundary case. Together they suggest that larger HEROS search budgets help downstream explanation quality when they also simplify the learned model. The MUX20 result does not imply that the task is inherently unsuitable for this translation setting; rather, under the budgets tested here, the learned models remain dense and non-ideal. The present results are also consistent with denser packets emerging from overlapping, overgeneral, or overspecific rules, but the current design does not isolate the separate effects of specified-variable count, rule overlap, and rule-set size.

Prompt design and audience conditioning yield a mixed but interpretable pattern. *Evidence+glossary* design generally encourages fuller verbalization of packet evidence, sometimes improving recall or lowering *causal overclaim rate*, but often at the cost of higher *hallucination rate* and longer outputs. Audience conditioning exposes distinct failure modes: layman outputs are safest but least complete, clinician outputs are most prone to overstatement, and expert outputs preserve the most evidence but invite the most unsupported elaboration. HEROS explains which matching rules support a prediction, but it does not by itself induce a higher-level symbolic summary of the full multiplexer relation. Richer expert-oriented summarization of recurring rule regularities is therefore a natural extension of the translation layer rather than a capability claimed here.

This study remains preliminary and has several limitations. It uses synthetic multiplexer benchmarks rather than biomedical datasets, relies on one seed and only two HEROS training profiles per dataset, and uses stratified 50-instance subsets for MUX11 and MUX20 rather than the full held-out folds. It also omits non-LLM presentation baselines and evaluates fidelity to the model-generated prediction-time evidence packet rather than to human-grounded causal evidence. The judge metrics remain proxy signals rather than participant-based outcomes, with additional caution warranted because an LLM from the same model family judged the generated explanations [12, 16]. The glossary intervention is also subjective: we intentionally used minimal feature-only entries to avoid leaking the true multiplexer mechanism, but this leaves glossary specificity unresolved.

Future work should add direct baselines, more HEROS checkpoints and seeds, systematic glossary-specificity tests, controlled synthetic problems that vary specified-variable count, overlap, density, and relational structure, real biomedical rule-learning tasks, perturbation-based faithfulness checks, and human evaluation where feasible [10, 11].

## 5 Code and Data Availability

Code, configurations, and run artifacts are available at https://github.com/UrbsLab/heros on the llm_exp branch.^1^

## 6 Conclusion

This paper presented a preliminary study of whether an LLM can restate prediction-time evidence from an evolutionary rule-based machine learning (ERBML) model for different readers. The ‘HEROS’ ERBML generated the evidence packets, so the contribution is a translation layer for ERBML prediction-time evidence rather than a new training method. Across all six runs, *evidence precision* and *prediction–Explanation agreement* remained perfect, while the larger differences arose from how completely prediction-time evidence was preserved, how cautiously it was verbalized, and how much technical detail was introduced for different readers. Under the budgets tested here, stronger HEROS training improved downstream explanation quality when it also simplified the selected rule model and evidence packet, as in MUX11, but not when the learned model remained dense and non-ideal, as in MUX20. The glossary-style prompt generally increased elaboration and sometimes improved audience-fit scores or lowered *causal overclaim rate*, but it often raised *hallucination rate* and increased output length. Audience customization also exposed stable trade-offs: layman outputs were safest, expert outputs preserved the most evidence, and clinician outputs were most prone to causal overstatement.

These results support a limited but useful conclusion: LLM-based verbalization of ERBML prediction-time evidence is feasible in a controlled preliminary setting, but its quality depends on the learned rule model and on how the evidence is presented to the generator. Immediate next steps are non-LLM baselines, broader ERBML training sweeps, controlled synthetic studies of rule density and specified-variable effects, and evaluation on real biomedical rule-learning problems.

## Supplementary Material

Supplementary Material

## Figures and Tables

**Table 1:** Metrics reported in the study. Rows explicitly marked as task-specific are introduced here. Judge metrics are rubric-based LLM outputs and should be interpreted as cautious proxies for audience fit.

Category	Metric	Calculation or operationalization	Range / direction	Basis
Faithfulness and Safety	*Evidence Precision*	∣*M* ∩ *E*∣/∣*M*∣, where *M* is the set of mentioned evidence features and *E* is the set of packet evidence features derived from the instance’s matching rules.	[0, 1]; higher is better.	Evidence attribution and rationale evaluation [7, 29].
Faithfulness and Safety	*Evidence Recall*	∣*M* ∩ *E*∣/∣*E*∣. Captures how much packet evidence is preserved in the explanation.	[0, 1]; higher is better.	Evidence attribution and rationale evaluation [7, 29].
Faithfulness and Safety	*Prediction–Explanation Agreement*	Mean of *A_i_*, where *A_i_* = 1 if the explanation either mentions the predicted class or mentions no class label explicitly, and *A_i_* = 0 otherwise.	[0, 1]; higher is better.	Task-specific metric introduced in this paper.
Faithfulness and Safety	*Rule Coverage*	For top-*k* supporting matching rules *K_i_*, computes $\frac{1}{|K_{i}|}\sum_{r\in K_{i}}1[F(r)\cap M_{i}\neq \emptyset ]$. Feature-overlap proxy only; not exact rule reconstruction.	[0, 1]; higher is better.	Task-specific coverage proxy introduced in this paper.
Faithfulness and Safety	*Hallucination Rate*	Mean of *H_i_*, where *H_i_* = 1 if an explanation contains unsupported features or unsupported reality claims.	[0, 1]; lower is better.	Hallucination construct [3]; implementation adapted to this pipeline.
Faithfulness and Safety	*Causal Overclaim Rate*	Mean of a binary flag for causal phrases or reality-claim phrases that go beyond packet evidence.	[0, 1]; lower is better.	Task-specific safety metric introduced in this paper.
Audience fit	*Audience Understandability*	Rubric-based LLM-judge score for whether the explanation is understandable for the requested audience.	[0, 1]; higher is better, but interpret cautiously.	Judge-based proxy motivated by XAI and LLM evaluation work [12, 13, 16].
Audience fit	*Audience Technical Fit*	Rubric-based LLM-judge score for whether the technical detail matches the requested audience.	[0, 1]; higher is better, but interpret cautiously.	Judge-based proxy motivated by XAI and LLM evaluation work [12, 13, 16].
Audience fit	*Flesch Reading Ease*	Standard readability formula computed for all audiences.	Higher means easier text; roughly 90–100 very easy, 60–70 standard, 30–50 difficult.	Readability formula [9].
Audience fit	*Flesch–Kincaid Grade Level*	Standard readability formula computed for all audiences.	Lower means simpler text; roughly 6 middle-school, 9–10 high-school, above 12 college-level.	Readability formula [14].
Miscellaneous	Word Count	Tokenized word count per explanation.	≥ 0; descriptive only.	Descriptive analysis choice in this paper.

**Table 2:** Dataset-level training-profile comparison. Each row summarizes one dataset under one HEROS training profile. *Evidence precision* and *prediction–explanation agreement* were 100.0 for all rows and are omitted. Judge scores are on a 0–1 scale and should be interpreted cautiously. Abbreviations: HEROS Acc. = HEROS test accuracy; Match Rules = mean matching rules per held-out instance; Ev. Recall = *Evidence Recall*; Halluc. = *Hallucination Rate*; Rule Cov. = *Rule Coverage*; COR = *Causal Overclaim Rate*; Aud. Und. = *Audience Understandability*; Aud. Tech. Fit = *Audience Technical Fit*.

Dataset	Training Profile	HEROS Acc.	Top Rules	Ideal %	Match Rules	Ev. Recall	Halluc.	Rule Cov.	COR	Aud. Und.	Aud. Tech. Fit	Words
MUX6	Minimal	100.0	8	100.0	1.00	100.0	16.0	100.0	0.0	0.902	0.873	90.78
MUX6	Extensive	100.0	8	100.0	1.00	100.0	18.3	100.0	0.0	0.888	0.868	90.42
MUX11	Minimal	81.2	56	18.8	4.86	94.4	5.7	99.9	17.0	0.827	0.833	126.11
MUX11	Extensive	98.8	24	87.5	2.24	99.4	18.3	100.0	9.0	0.842	0.846	102.73
MUX20	Minimal	60.1	54	0.0	12.34	73.7	12.0	94.3	32.0	0.796	0.818	139.74
MUX20	Extensive	63.2	69	0.0	12.88	68.6	8.7	93.0	34.0	0.813	0.829	141.01

**Table 3:** Illustrative MUX11 held-out example (InstanceID 287) under *evidence-only prompt* based run. The same held-out instance yields different HEROS packets under the two training profiles. Within each block, audience rows are ordered *Layman, Clinician*, and *Expert*. The table can be read vertically for audience effects and horizontally for training-profile and packet effects. Column Hall. reports whether the hallucination detector flagged the row; the italicized span in quotation marks the flagged wording in the minimal expert row.

Training Profile	Audience	Hall.	Words	Output
**Minimal packet summary:** The selected HEROS model predicts **class 1** with probability **0.665**. Three matching rules support the prediction and no conflicting matching rules are present. The strongest recurring feature pattern is *A*_1_ = 0 with *R*_1_ = 1, together with *A*_2_ = 0 and either *R*_4_ = 1 or *R*_5_ = 1.				
*Minimal*	Layman	No	86	“The model leaned toward **class 1** because several conditions in the data all support this prediction. Specifically, the values show that **A_2 = 0**, and combinations of **A_1 = 0** with **R_1 = 1** and **R_4 = 1**, as well as **A_0 = 0** and **A_1 = 0** with **R_1 = 1** and **R_5 = 1**, all point to class 1. Since all the active rules agree, the model is confident in this choice. Keep in mind, this explanation reflects how the model made its decision, not a statement of fact.”
*Minimal*	Clinician	No	127	“The prediction of **class 1** with moderate confidence (about **66%**) is supported by three active rules that all agree on this outcome. One rule indicates that when **A_2 = 0**, class 1 is predicted, though this rule alone has mixed support with about 50% of similar cases in training being class 1. Two additional rules, which consider combinations of **A_1, R_1, R_4**, and **R_5** values, provide stronger support with around 74–75% of matched training instances belonging to class 1. Specifically, the presence of **R_1 = 1, R_4 = 1**, and **R_5 = 1** alongside **A_0 = 0** and **A_1 = 0** aligns with these more confident rules. Overall, the evidence pattern from these rules consistently supports the prediction, but this explanation reflects model reasoning rather than a definitive causal conclusion.”
*Minimal*	Expert	Yes	164	“The prediction of **class 1** with moderate confidence (**probability 0.665**) is supported by three active rules, all of which favor class 1 without conflict. **Rule 8051** matches on **A_2 = 0** and has a "*near-even*" class distribution in training (about 50% class 1), contributing modestly to the vote. **Rules 7904** and **7831** provide stronger support: both require **A_1 = 0** and **R_1 = 1**, with Rule 7904 also requiring **R_4 = 1** and Rule 7831 requiring **A_0 = 0** and **R_5 = 1**. These latter two rules matched fewer instances but had higher accuracy (about 74–75%) and fitness, indicating more reliable support for class 1. The instance’s feature values (**A_0 = 0, A_1 = 0, A_2 = 0, R_1 = 1, R_4 = 1, R_5 = 1**) satisfy all three rules’ conditions, leading to unanimous agreement among the active rules. This consensus underpins the moderate confidence in the class 1 prediction. This explanation reflects the model’s reasoning based on rule matches and training support, not a causal inference or ground truth statement.”
**Extensive packet summary:** The selected HEROS model predicts **class 0** with probability **0.775**. Three matching rules are present, of which one strongly supports the prediction and two contradict it. The decisive supporting rule uses *A*_0_ = 0, *A*_1_ = 0, *A*_2_ = 0, and *R*_0_ = 0; the conflicting rules additionally involve *R*_2_ = 1 together with either *R*_3_ = 0 or *R*_6_ = 0.				
*Extensive*	Layman	No	87	“The model leaned toward **class 0** mainly because one rule matched that supports this prediction, based on the values of **A_0 = 0, A_1 = 0, A_2 = 0**, and **R_0 = 0**. However, two other rules also matched that suggest a different class, since they rely on **A_0 = 0, A_2 = 0, R_2 = 1**, and either **R_3 = 0** or **R_6 = 0**. This means the evidence is mixed, but overall the model favored class 0. Keep in mind this explanation reflects how the model made its decision, not a definitive fact.”
*Extensive*	Clinician	No	118	“The prediction of **class 0** is mainly supported by one strong rule that matches the instance’s feature values **A_0 = 0, A_1 = 0, A_2 = 0**, and **R_0 = 0**, which had nearly 100% class 0 support in training. However, two other active rules, which also match the instance’s features including **R_2 = 1** and **R_3 = 0** or **R_6 = 0**, predict class 1 with moderate support around 79%. This creates some conflicting evidence, but since the strongest rule supports class 0 and most active rules lean that way, the model predicts class 0 with moderate confidence of about **77%**. This explanation reflects the model’s reasoning based on the matched rules and feature values, not a definitive statement of truth.”
*Extensive*	Expert	No	142	“The prediction of **class 0** with moderate confidence (**probability 0.775**) is primarily supported by **Rule 88893**, which matches the instance features **A_0 = 0, A_1 = 0, A_2 = 0**, and **R_0 = 0**. This rule has strong support, perfect accuracy on 266 training instances, and contributes exclusively to class 0 votes. However, two conflicting rules (**90750** and **91623**) also match the instance, both predicting class 1 with moderate support and about 79% accuracy on their respective training subsets. These conflicting rules match on **A_0 = 0, A_2 = 0, R_2 = 1**, combined with **R_6 = 0** or **R_3 = 0**, and contribute predominantly to class 1 votes. The overall evidence is mixed, with the majority of active rules favoring class 0 but notable opposition from class 1 rules. This explanation reflects the model’s reasoning based on rule matches and does not imply causal relationships or ground truth.”

**Table 4:** Cross-dataset prompt comparison under both HEROS training profiles. Rows compare *evidence-only* and *evidence+glossary* prompt designs after aggregating across datasets. *Evidence precision* and *prediction–explanation agreement* are omitted. Judge scores are on a 0–1 scale and should be interpreted cautiously. Percentage-style metrics are shown as percentages. Abbreviations: Ev. Recall = *Evidence Recall*; Halluc. = *Hallucination Rate*; Rule Cov. = *Rule Coverage*; COR = *Causal Overclaim Rate*; Aud. Und. = *Audience Understandability*; Aud. Tech. Fit = *Audience Technical Fit*; FRE = *Flesch Reading Ease*; FKGL = *Flesch–Kincaid Grade Level*.

Training Profile	Setting	Ev. Recall	Halluc.	Rule Cov.	COR	Aud. Und.	Aud. Tech. Fit	FRE	FKGL	Words
Minimal	Evidence-Only	88.6	10.2	97.7	16.4	0.841	0.841	56.75	10.67	116.28
Minimal	Evidence+Glossary	90.2	12.2	98.4	16.2	0.843	0.842	55.70	10.83	121.47
Extensive	Evidence-Only	89.2	14.2	97.7	15.8	0.846	0.848	56.96	10.46	109.10
Extensive	Evidence+Glossary	89.4	16.0	97.6	12.9	0.849	0.848	55.97	10.73	113.67

**Table 5:** Cross-dataset audience comparison under both HEROS training profiles. Rows compare *layman, clinician*, and *expert* after aggregating across datasets. *Evidence precision* and *prediction–explanation agreement* are omitted. Judge scores are on a 0–1 scale and should be interpreted cautiously. Percentage-style metrics are shown as percentages. Abbreviations are the same as in Table 4.

Training Profile	Setting	Ev. Recall	Halluc.	Rule Cov.	COR	Aud. Und.	Aud. Tech. Fit	FRE	FKGL	Words
Minimal	Layman	86.6	2.7	97.1	4.3	0.780	0.691	54.78	10.82	82.97
Minimal	Clinician	88.5	3.0	97.9	32.3	0.839	0.876	52.60	11.91	121.51
Minimal	Expert	93.0	28.0	99.2	12.3	0.906	0.958	61.30	9.53	152.16
Extensive	Layman	86.2	6.7	97.1	3.0	0.786	0.691	55.72	10.71	79.33
Extensive	Clinician	88.4	3.0	97.2	29.3	0.855	0.881	52.47	11.77	114.51
Extensive	Expert	93.3	35.7	98.7	10.7	0.902	0.972	61.20	9.31	140.32

## CCS Concepts

• **Computing methodologies** → **Machine learning**; *Rule learning*; Natural language generation; • **Human-centered computing** → *Natural language interfaces*.

## Footnotes

1 Prompt templates: evaluation/experiments/heros_llm/prompt_templates.py. Judge code: evaluation/experiments/heros_llm/judge.py. Partitioned MUX datasets: evaluation/datasets/partitioned/multiplexer/.
