# Supplementary material for: Audience-Customized Translation of Rule-Based Evidence with Large Language Models Across Multiplexer Benchmarks
Source: Genet Evol Comput Conf. Author manuscript; Available in PMC 2026 Sep 1. (PMC13528409; doi:10.1145/3795101.3814722)
Supplement: Supplementary file 1 [file NIHMS2205433-supplement-Supplementary_Material.pdf]

# Supplementary Material for Audience-Customized Translation of Rule-Based Evidence with Large Language Models Across Multiplexer Benchmarks

Harsh Bandhey[1], Gabriel Lipschutz-Villa[2], Khoi Dinh[2], Malek Kamoun[2], and Ryan J. Urbanowicz*[1]
Cedars Sinai Health Sciences University[1]; University of Pennsylvania[2]

## 1 Supplementary Tables

This supplement provides supporting detail for the main paper. The main manuscript retains the full argument, the metric definitions, the primary result tables, and the qualitative example discussed in the Results section. The material collected here is limited to reproducibility-oriented setup information, prompt and judge documentation, and one illustrative example of the ideal-MUX6 reference table used to clarify the HEROS prediction mechanics described in Methods. It does not add new empirical claims. The study used versioned prompt templates and a separate judge rubric that are implemented in `evaluation/experiments/heros_llm/prompt_templates.py`, `evaluation/experiments/heros_llm/prompt_builder.py`, and `evaluation/experiments/heros_llm/judge.py` on the `llm_exp` branch. Tables here summarize the parts of those prompts that are most important for interpreting the experiments.

**Supplementary Table S1.** Prompt structure used in the final study. The code labels `condition_b` and `condition_c` correspond to *evidence-only* and *evidence+glossary* prompt designs in the paper.

| **Component** | **Implemented behavior** |
|---|---|
| Shared system prompt (`v2_minimal`) | Explain only one prediction, use only the supplied prediction-time evidence packet, do not use the underlying algorithm name, do not add domain meaning or unseen features, describe model behavior rather than reality, acknowledge conflict when present, and end with a brief limitation sentence. |
| Layman addon | Plain language, feature names exactly as given, no rule IDs, no metadata counts, and no numeric probabilities. |
| Clinician addon | Concise feature–value reasoning with moderate technical detail, no rule IDs, and optional training-support summaries when provided. |
| Expert addon | Technical wording that may include rule IDs, exact probabilities, conflict structure, and detailed rule metadata when provided. |
| Evidence-only (`condition_b`) | User prompt includes predicted class, confidence wording, evidence summary, relevant instance feature values, and active rules, but no feature definitions. |
| Evidence+glossary (`condition_c`) | Uses the same packet fields as the Evidence-only prompt and additionally includes concise feature definitions for rule-relevant features; these definitions are used only to clarify labels, not to infer extra domain meaning. |

**Supplementary Table S2.** Judge-rubric structure used in the final study.

| **Component** | **Implemented behavior** |
|---|---|
| Judge system prompt (`v2`) | Strictly evaluate the explanation against the supplied packet, intended audience, and stated constraints. |
| Judge inputs | Audience, experiment condition, predicted class, agreement status, evidence-strength label, and the generated explanation text. |
| Judge outputs | JSON containing `audience_understandability_score`, `audience_technical_fit_score`, and brief `judge_notes`. |
| Judge scoring rules | Score understandability for the intended audience, score technical fit for the intended audience, penalize added domain meaning beyond the packet evidence, and return JSON only. |
| Artifact writing | The pipeline writes prompt rows, judge-request rows, and judge-result rows for completed runs when prompt writing and judge scoring are enabled in the configuration. |

**Supplementary Table S3.** Benchmark configuration with mean matching rules (MMR) from the final runs. Each benchmark contributes 50 evaluated held-out instances under two HEROS training profiles, producing 600 generated explanations per dataset and 1,800 explanations overall.

| Dataset | Train | Held-out Test | Evaluated per Training Profile | MMR Minimal | MMR Extensive |
|---|---|---|---|---|---|
| MUX6 | 450 | 50 | 50 | 1.00 | 1.00 |
| MUX11 | 4,500 | 500 | 50 | 4.86 | 2.24 |
| MUX20 | 9,000 | 1,000 | 50 | 12.34 | 12.88 |

**Supplementary Table S4.** Major HEROS training and model-selection settings used across MUX6, MUX11, and MUX20.

| Setting | Minimal | Extensive |
|---|---|---|
| Outcome type | `class` | `class` |
| Excluded columns | `Group`, `InstanceID` | `Group`, `InstanceID` |
| Rule-learning iterations | 10,000 | 100,000 |
| Rule population size | 500 | 1,000 |
| Model-level iterations | 40 | 100 |
| Model population size | 100 | 100 |
| Crossover probability | 0.8 | 0.8 |
| Mutation probability | 0.04 | 0.04 |
| Fitness function | `pareto` | `pareto` |
| Subsumption | `both` | `both` |
| Compaction | `sub` | `sub` |
| Random seed | 42 | 42 |
| Target model selection | `auto_select_top_model` | `auto_select_top_model` |

**Supplementary Table S5.** LLM explanation and evaluation run parameters used in the final study.

| Setting | Value |
|---|---|
| Prompt version | `v2_minimal` |
| Training profiles | Minimal; Extensive |
| Prompt Design | Evidence-only and Evidence+glossary |
| Audiences | Layman, Clinician, Expert |
| Sampling strategy | Stratified by prediction and rule-match bucket, with the full MUX6 test fold used when available |
| Generation model | `gpt-4.1-mini` |
| Generation temperature | 0.0 |
| Generation max tokens | 400 |
| Judge model | `gpt-4.1-mini` |
| Judge scoring | Applied to all completed runs; post hoc for minimal-profile runs |
| Judge temperature | 0.0 |
| Judge max tokens | 300 |
| Prompt metadata exposure | Clinician and Expert only |
| Glossary exposure | Experiment 2 only; minimal feature-only glossary |

**Supplementary Table S6.** Ideal MUX6 rule set and illustrative prediction example. The ideal MUX6 solution contains eight fully specific rules: for each address-bit pair $(A_0, A_1)$, the selected register bit determines the class. The example matches the idealized discussion in the main paper's HEROS prediction subsection.

| Rule | Address Bits | Selected Register Bit | Class |
|---|---|---|---|
| $r_1$ | $A_0 = 0,\ A_1 = 0$ | $R_0 = 0$ | 0 |
| $r_2$ | $A_0 = 0,\ A_1 = 0$ | $R_0 = 1$ | 1 |
| $r_3$ | $A_0 = 0,\ A_1 = 1$ | $R_1 = 0$ | 0 |
| $r_4$ | $A_0 = 0,\ A_1 = 1$ | $R_1 = 1$ | 1 |
| $r_5$ | $A_0 = 1,\ A_1 = 0$ | $R_2 = 0$ | 0 |
| $r_6$ | $A_0 = 1,\ A_1 = 0$ | $R_2 = 1$ | 1 |
| $r_7$ | $A_0 = 1,\ A_1 = 1$ | $R_3 = 0$ | 0 |
| $r_8$ | $A_0 = 1,\ A_1 = 1$ | $R_3 = 1$ | 1 |

**Illustrative example.** Consider the instance $A_0 = 1$, $A_1 = 0$, $R_0 = 1$, $R_1 = 0$, $R_2 = 1$, $R_3 = 1$. Under the ideal rule set, only $r_6$ matches, so the match set is $M = \{r_6\}$. If numerosity is 1 for every ideal rule, then the class vote totals are $V(0) = 0$ and $V(1) = 1$. After normalization, $P(0) = 0.0$ and $P(1) = 1.0$, so the predicted class is 1. This illustrates the minimal single-rule case against which denser learned HEROS match sets can be contrasted.
